# Complete Genome Sequence of Methylomonas denitrificans Strain FJG1, an Obligate Aerobic Methanotroph That Can Couple Methane Oxidation with Denitrification

**DOI:** 10.1128/genomeA.00276-18

**Published:** 2018-04-26

**Authors:** Fabini D. Orata, K. Dimitri Kits, Lisa Y. Stein

**Affiliations:** a Department of Biological Sciences, University of Alberta, Edmonton, Alberta, Canada; b Department of Chemical and Materials Engineering, University of Alberta, Edmonton, Alberta, Canada; c Department of Microbiology and Ecosystem Science, Division of Microbial Ecology, University of Vienna, Vienna, Austria

## Abstract

Methylomonas denitrificans strain FJG1 is a member of the gammaproteobacterial methanotrophs. The sequenced genome of FJG1 reveals the presence of genes that encode methane, methanol, formaldehyde, and formate oxidation. It also contains genes that encode enzymes for nitrate reduction to nitrous oxide, consistent with the ability of FJG1 to couple denitrification with methane oxidation.

## GENOME ANNOUNCEMENT

The genus *Methylomonas* belongs to the class Gammaproteobacteria, order Methylococcales, and family Methylococcaceae (1). Members of this genus are able to utilize methane as their sole source of carbon and energy (2). Methane is a common low-value industrial by-product (3) and is a more potent greenhouse gas than carbon dioxide (4). Methanotrophs play a key role in the global carbon cycle (5) and can be used to convert methane into value-added products such as biofuels and biopolymers (3). Here, we report the complete genome sequence of *Methylomonas denitrificans* strain FJG1, a recently described methanotrophic species (6).

Genomic DNA from *M. denitrificans* strain FJG1 was extracted using the MasterPure complete DNA and RNA purification kit (Epicentre). Sequencing libraries were then prepared using the RS II SMRTbell template preparation kit 1.0 (Pacific Biosciences) and sequenced with the P6 v2 single-molecule real-time (SMRT) sequencing platform (Pacific Biosciences). The 300,584 raw reads resulted in 106,388 quality-filtered trimmed reads, which were subsequently assembled *de novo* using the Hierarchical Genome Assembly Process of the SMRT Analysis software 2.2 (7). The resulting genome is 5,172,098 bp in size, with a G+C content of 51.7% and a coverage of 228×. Whole-genome comparisons (8, 9) of FJG1 with other *Methylomonas* strains reveal its highest similarity with Methylomonas methanica NCIMB 11130 (10).

The genome of FJG1 was annotated using the Prokaryotic Genome Annotation Pipeline 4.1 (11) to reveal 4,559 protein-coding genes, 3 rRNA operons (16S, 23S, and 5S), 47 tRNAs, and 4 noncoding RNAs (ncRNAs). The aerobic oxidation of methane to methanol is catalyzed by methane monooxygenase (2). Strain FJG1 contains the operons *pmoCAB* and *pxmABC*, which encode particulate methane monooxygenase (pMMO) and copper-containing membrane monooxygenase (12). However, the soluble methane monooxygenase operon (*mmoXYBZDCGR*) (13) is not present. The genome also contains genes encoding cyanoglobins (*hbN*), one of which is upregulated under hypoxic conditions and hypothesized to bind oxygen for delivery to pMMO (6). Methanol is converted to formaldehyde by pyrroloquinoline quinone (PQQ)-dependent methanol dehydrogenases (2). Gene clusters for methanol dehydrogenases (*mxaDFJGIRSACKL* and *xoxFJ*) and PQQ biosynthesis (*pqqABCDE* and *pqqFG*) are present. Also present are genes involved in formaldehyde oxidation, including those for the tetrahydromethanopterin (*fae*, *fhcCDAB*, *mch*, *mptG*, and *mtdB*) and tetrahydrofolate (*fch*, *fhs*, and *mtdA*) pathways and an NAD-dependent formate dehydrogenase (*fdsABGCD*) for formate oxidation. Additionally, genes involved in C1 assimilation through the ribulose monophosphate, tricarboxylic acid, Embden-Meyerhof-Parnas, and Entner-Doudoroff pathways were identified.

FJG1 possesses genes for nitrogen acquisition, including ammonium (*amtB*), nitrate (*narK* and *nrtA*), and urea (*urtABCDE*) transporters, and urease genes (*ureABCDEFG*). Genes for denitrification, namely, nitrate (*narGHJI* and *napABC*), nitrite (*nirK* and *nirS*), and nitric oxide (*norCB*) reductases, are also present. The strain contains alanine dehydrogenase (*aldA*) for the assimilation of ammonium by reductive amination of pyruvate (2). Assimilatory nitrate (*nasA*) and nitrite (*nirBD*) reductases are also present. In contrast, under ammonium-limiting conditions, ammonia is assimilated through the glutamine synthetase (*glnA*)-glutamate synthase (*gltB*) pathway (2), both of which are also present in FJG1.

### Accession number(s).

The whole-genome sequence of M. denitrificans FJG1 has been deposited in GenBank under the accession number CP014476. The version described in this paper is the first version, CP014476.1.
